# Association of Endothelial Activation and Stress Index with Prognosis in Posterior Circulation Infarcts Treated with Recanalization Therapy

**DOI:** 10.3390/diagnostics15243234

**Published:** 2025-12-17

**Authors:** Deniz Kamaci Sener, Cemile Haki, Gulcin Koc Yamanyar, Fatma Nur Kandemir, Suat Kamisli, Kaya Sarac

**Affiliations:** 1 Department of Neurology, Bursa City Hospital, Bursa 16110, Turkey; cemilehaki@gmail.com (C.H.); dr.gulcinkoc@hotmail.com (G.K.Y.); fatmanurkandemir.1263@gmail.com (F.N.K.);; 2 Department of Radiology, Bursa City Hospital, Bursa 16110, Turkey

**Keywords:** EASIX, reperfusion therapy, acute ischemic stroke, endothelial dysfunction, posterior circulation

## Abstract

**Background**: Endothelial dysfunction plays a critical role in ischemic stroke. The Endothelial Activation and Stress Index (EASIX), calculated from creatinine, lactate dehydrogenase (LDH), and platelet levels, reflects endothelial injury. This study aimed to investigate the relationship between EASIX and 90-day mortality in patients with posterior circulation ischemic stroke (PCIS) treated with mechanical thrombectomy. **Methods**: Fifty-eight patients with acute ischemic stroke who underwent mechanical thrombectomy (MT) or MT combined with intravenous thrombolysis (intravenous tissue plasminogen activator (tPA)) for posterior circulation ischemic stroke (PCIS) were included. EASIX was calculated using 24 h laboratory values of creatinine, LDH, and platelets. Its association with 90-day mortality, length of hospital stay, intubation, and parenchymal hemorrhage was analyzed. **Results**: In patients receiving reperfusion therapy, the Endothelial Activation and Stress Index (EASIX) showed modest ability to predict 90-day mortality (AUC = 0.583, 95% CI 0.428–0.739, *p* = 0.295). Higher EASIX values were linked to a 6.58-fold increase in mortality risk. Patients with elevated EASIX were generally older, had more frequent hyperlipidemia, had higher 24 h National Institutes of Health Stroke Scale (NIHSS) scores, had greater need for intubation, and had higher in-hospital mortality. **Conclusions**: EASIX is a simple, inexpensive, and non-invasive marker that may reflect endothelial dysfunction and help predict mortality in PCIS patients undergoing reperfusion therapy. Higher EASIX values are associated with poorer prognosis. Early identification of high-risk patients may support secondary prevention strategies.

## 1. Introduction

Ischemic stroke is one of the leading causes of death and disability worldwide, resulting in approximately 5 million deaths annually [1]. Approximately 20% of strokes occur in the vertebrobasilar system, known as the posterior circulation [2]. Despite improved recanalization rates with mechanical thrombectomy (MT) and tissue plasminogen activator (tPA), posterior circulation ischemic stroke (PCIS) continues to have a poor prognosis (68–90%) [3]. Although several studies have demonstrated that mechanical thrombectomy (MT) is safe and effective in posterior circulation ischemic stroke (PCIS), the BEST trial reported no significant superiority of MT over standard medical treatment [4,5].

Endothelial dysfunction, which contributes to vascular injury and reperfusion failure, plays a critical role in the pathophysiology of ischemic stroke [6]. Following acute ischemic stroke (AIS) and reperfusion therapy, the generation of reactive oxygen species (ROS) increases markedly, triggering inflammation and secondary vascular injury [7]. The vascular endothelium acts as a dynamic organ that maintains vascular homeostasis and regulates tone, permeability, and leukocyte adhesion [8]. Excessive ROS production may lead to endothelial dysfunction, a prothrombotic and proinflammatory condition characterized by vasoconstriction, inflammation, and vascular remodeling [9,10]. Several biomarkers, including integrins, selectins, cadherins, homocysteine, interleukin (IL)-8, IL-18, and C-reactive protein (CRP), have been proposed to reflect endothelial dysfunction. Among these, CRP has been consistently associated with endothelial injury and poor outcomes in both cardiovascular disease and ischemic stroke [11,12,13,14]. However, most studies have focused on anterior circulation infarctions, and the prognostic role of endothelial biomarkers in PCIS remains largely underexplored. This knowledge gap may stem from the relative rarity of PCIS, which limits the sample sizes and statistical power of prospective investigations.

Existing prognostic tools, such as the NIHSS and APACHE II scores, are valuable but have limited applicability to posterior circulation ischemic stroke (PCIS). Biomarkers like CRP and N-terminal pro-brain natriuretic peptide (NT-proBNP) have also demonstrated prognostic potential; however, their use in clinical practice is restricted by cost, limited specificity, and variable availability [15,16,17].

The Endothelial Activation and Stress Index (EASIX), defined by Luft et al., is calculated from creatinine, lactate dehydrogenase (LDH), and platelet count. Each parameter has a mechanistic association with endothelial injury: creatinine reflects renal and endothelial dysfunction, LDH indicates cellular stress and inflammation, and platelet count represents thrombotic activity. Together, these components provide an integrated estimate of endothelial stress. EASIX was initially developed in patients with hematologic malignancies and transplantation-related endothelial injury, where it demonstrated a strong correlation with survival [18]. More recently, Kleeberg et al. proposed that EASIX could serve as a prognostic biomarker in acute ischemic stroke [19]. Similarly, Huang et al., analyzing data from 43,853 individuals in the NHANES cohort, reported that elevated EASIX was positively associated with both stroke prevalence and all-cause mortality, even after adjustment for multiple covariates [20]. These findings highlight the potential role of EASIX in cerebrovascular disease; however, its prognostic value in posterior circulation ischemic stroke (PCIS), particularly in patients undergoing reperfusion therapy, has not yet been established. Therefore, this study aimed to evaluate the predictive value of EASIX for 90-day mortality in patients with PCIS treated with mechanical thrombectomy (MT) or MT combined with intravenous thrombolysis (tPA).

## 2. Materials and Methods

This study included 58 patients who presented to the emergency department of Bursa City Hospital with acute ischemic stroke (AIS) between March 2020 and November 2024. Posterior circulation occlusion was confirmed using carotid computed tomography (CT) angiography and cranial CT angiography. All patients underwent mechanical thrombectomy (MT) within 6 h or MT combined with intravenous thrombolysis (tPA) within 4.5 h after symptom onset and were subsequently monitored in the intensive care unit. In accordance with our institutional protocol for posterior circulation ischemic stroke, only patients treated with MT or MT + tPA were included to achieve a homogeneous cohort. Patients receiving other treatment modalities were excluded. There were no missing laboratory data for creatinine, LDH, or platelet count in the analyzed cohort.

Demographic and clinical characteristics, laboratory data, and radiological imaging of the patients were retrospectively reviewed from patient files. The following variables were recorded: age, sex, smoking and alcohol use, hypertension (HT), diabetes mellitus, cardiac disease, history of prior stroke or transient ischemic attack (TIA), and presence of atrial fibrillation. Stroke severity at hospital admission was assessed using the National Institutes of Health Stroke Scale (NIHSS) (0 h NIHSS). Furthermore, stroke etiology was classified according to the Trial of Org 10172 in Acute Stroke Treatment (TOAST). Symptom onset time, hospital arrival time (symptom-to-door time), presence of wake-up stroke, and time from symptom onset to reperfusion therapy initiation (symptom-to-groin time) were also documented. Recanalization success during mechanical thrombectomy (MT) was classified using the Thrombolysis in Cerebral Infarction (TICI) scale. Successful revascularization was defined as TICI 2b–3.

Post-procedure 24 h NIHSS (24 h NIHSS), Acute Physiology and Chronic Health Evaluation (APACHE) II score, hospital stay duration, intubation status (yes/no), duration of intubation (days), post-MT decompression, hemorrhagic transformation (symptomatic or asymptomatic) on 24 h post-MT/MT + tPA brain CT, and 90-day modified Rankin Scale (mRS) scores were recorded. The 90-day mRS was determined during outpatient clinic visits. Poor prognosis was defined as death within 90 days. Patients with mRS 0–2 were classified as independent, mRS 3–5 as dependent, and mRS 6 as deceased. For deceased patients, outcome information was obtained through telephone interviews with relatives.

Platelet count and creatinine levels were obtained at the time of emergency department admission, prior to any reperfusion therapy. In our institution, serum LDH is routinely measured after the endovascular procedure, during the patient’s initial admission to the intensive care unit (ICU). In the original version of the manuscript, these parameters were collectively described as “within 24 h,” and this has now been clarified to accurately reflect the timing of each laboratory measurement.

The Endothelial Activation and Stress Index (EASIX) was calculated using the following formula:

**EASIX = LDH [U/L] × creatinine [mg/dL]/platelet count [10^9^/L].**

Patients with systemic inflammatory or hematologic diseases, immunosuppressant therapy, active malignancy, recent major surgery or trauma (within the past 3 months), or those lost to follow-up within 90 days after acute ischemic stroke (AIS) were excluded from the analysis.

This study was conducted retrospectively using anonymized clinical data. Because of the retrospective design and the nature of the data, individual informed consent was not required according to local ethical standards and institutional policies.

### Statistical Analysis

Descriptive statistics for continuous variables were presented as median and interquartile range (IQR), while categorical variables were given as numbers and percentages. The normality of the data was checked with the Shapiro–Wilk test. Comparisons between independent groups without normal distribution were performed using the Mann–Whitney U test, and relationships between categorical variables were analyzed with Pearson’s chi-square or Fisher’s exact test. Receiver operating characteristic (ROC) curve analysis was performed to evaluate the ability of EASIX to predict mortality and to determine the optimal cut-off value, defined as the point with the shortest distance to the upper-left corner of the curve. The area under the curve (AUC), sensitivity, specificity, positive predictive value (PPV), and negative predictive value (NPV) were reported. Variables such as age, sex, clinical and laboratory findings, and treatment types, as well as other possible factors affecting mortality, were first examined using univariate logistic regression. Variables that were statistically significant in the univariate analysis were included in a multivariate logistic regression model (Enter method). A two-tailed *p* value < 0.05 was considered statistically significant. All analyses were carried out using SPSS for Windows, version 25 (SPSS Inc., Chicago, IL, USA).

## 3. Results

The study included 58 patients diagnosed with PCIS who underwent reperfusion therapy. Of the patients, 74.1% were male and 25.9% female. The median age was 70 (Q1–Q3: 57–76). Other demographic data are presented in Table 1.

**Figure 1:**
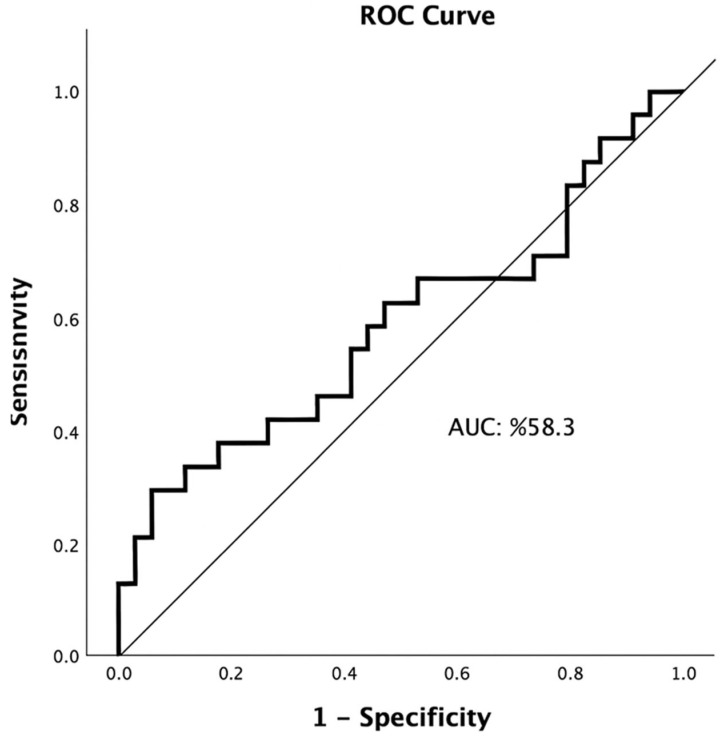
Receiver operating characteristic (ROC) curve illustrating the predictive performance of the Endothelial Activation and Stress Index (EASIX) for 90-day mortality (AUC = 0.583, 95% CI: 0.428–0.739).

The optimal EASIX cut-off value for predicting 90-day mortality (mRS 6) was ≥ 1.63, with an AUC of 0.583 (95% CI: 0.428–0.739, *p* = 0.295). At this threshold, sensitivity was 29.2%, specificity was 94.2%, positive predictive value (PPV) was 77.8%, and negative predictive value (NPV) was 65.3%. These findings indicate that EASIX has high specificity but limited sensitivity in predicting 90-day mortality. (Table 2; Figure 1).

Patients were stratified into two groups based on EASIX values (<1.63 vs. ≥1.63). Significant differences between the groups were observed in age, presence of hyperlipidemia, intubation, in-hospital mortality, 24 h NIHSS, discharge mRS, and 90-day mRS (all *p* < 0.05). The high-EASIX group had a significantly higher proportion of patients aged > 70 years (*p* = 0.011), hyperlipidemia (*p* = 0.009), intubation (*p* = 0.035), in-hospital mortality (*p* = 0.016), 24 h NIHSS (*p* = 0.049), and both discharge and 90-day mRS (*p* = 0.048 for each) (Table 3).

Other variables, including smoking and heart disease, did not differ significantly between groups. This lack of statistical significance is most likely due to the limited sample size and heterogeneity of the study population. These factors may have reduced statistical power and obscured potential associations.

Univariate and multivariate logistic regression analyses were performed to evaluate the clinical parameters affecting mortality. In the univariate analysis, a high EASIX value was significantly associated with mortality (*p* = 0.028). Patients with high EASIX had a 6.588-fold increased risk of death (95% CI: 1.230–35.277). Intubation status (yes/no) was also a strong predictor, being associated with a 12.833-fold increased risk of mortality. Additionally, an intubation duration of >3 days was associated with a 6.133-fold higher mortality risk. The presence of hemorrhage (11.875-fold), APACHE II > 13 (7.381-fold), NIHSS > 12 on admission (3.510-fold), 24 h NIHSS > 15 (6.571-fold), and discharge mRS > 5 (125.4-fold) were all associated with substantially increased mortality risk. No independent predictors were identified in the multivariate logistic regression model (Table 4).

## 4. Discussion

In this retrospective study, we found that a high Endothelial Activation and Stress Index (EASIX)—reflecting endothelial dysfunction and systemic stress—was significantly associated with both in-hospital and 90-day mortality in patients with posterior circulation ischemic stroke (PCIS) who underwent reperfusion therapy. Specifically, patients with high EASIX values had a 6.58-fold higher risk of 90-day mortality. In addition, elevated EASIX was correlated with older age, hyperlipidemia, higher 24 h NIHSS scores, and a greater need for intubation.

EASIX has been recognized as a valuable biomarker reflecting endothelial dysfunction and clinical prognosis across various patient populations. Elevated EASIX levels have been linked to poor survival outcomes in multiple myeloma and diffuse large B-cell lymphoma [21,22]. In a prospective study of allogeneic stem cell transplant recipients, a pre-transplant EASIX value ≥ 3 was associated with a twofold increase in treatment-related mortality [23]. Similarly, in critically ill patients with COVID-19, high EASIX values were shown to predict in-hospital mortality [24].

Several studies have explored the relationship between coronary artery disease and EASIX. In a study involving 1934 patients with acute coronary syndrome, EASIX was identified as a prognostic marker for overall survival before and after cardiac catheterization [25]. Sang et al. reported that EASIX outperformed its individual components (creatinine, LDH, and platelet count) in predicting 30-day mortality following acute myocardial infarction (AMI) [26]. In line with these findings, our study also demonstrated a significant association between elevated EASIX values and mortality in patients with posterior circulation ischemic stroke (PCIS) who underwent reperfusion therapy.

EASIX combines three well-established mortality predictors in AIS—creatinine, LDH, and platelet count—into a single index. Serum creatinine reflects renal function, and elevated creatinine levels indicate renal dysfunction, which is closely linked to cerebral small-vessel disease [27]. The kidney and brain share delicate vascular structures exposed to high pulsatile flow and pressure with low resistance [28]. Advanced age, HT, diabetes, smoking, and dyslipidemia are common risk factors for ischemic stroke and renal dysfunction. Oxidative stress, inflammation, and accumulation of uremic toxins further impair renal function, aggravating endothelial dysfunction and atherosclerosis [29]. Elevated serum creatinine is a strong independent predictor of post-stroke survival in the elderly [30].

Dehydration is considered to play a multifactorial role in the pathophysiology of ischemic stroke. It increases blood viscosity and may reduce cerebral perfusion by decreasing intravascular volume. Elevated hematocrit levels have been associated with larger infarct volumes, supporting the link between dehydration and impaired cerebral hemodynamics. Moreover, dehydration has been correlated with thrombotic complications, such as recurrent embolic stroke and venous thromboembolism following acute stroke [31]. In patients treated with intravenous thrombolysis (tPA), dehydration was associated with unfavorable outcomes, particularly among those with NIHSS scores greater than 6 [32]. Consistently, Dong et al. reported that patients with a glomerular filtration rate (GFR) below 60 mL/min/1.73 m^2^ had a fourfold higher risk of death at 3 months compared to those with GFR values above 90 mL/min/1.73 m^2^ [33]

In acute ischemic stroke (AIS), endothelial injury triggers platelet activation and aggregation at the site of vascular damage, promoting thrombosis and increased platelet consumption. Activated platelets contribute to thromboinflammation, amplifying tissue injury and the inflammatory cascade. A reduced platelet count may result from enhanced platelet consumption or excessive destruction, both of which are observed in severe systemic inflammation and disseminated intravascular coagulation. Platelet count has been linked to mortality, stroke recurrence, and poor functional outcomes in several studies [34,35].

LDH is a glycolytic enzyme commonly found in human tissues and is released from cells when the cell membrane is damaged [36]. In AIS, disruption of cerebral blood flow leads to hypoxia and necrosis. As a result of tissue damage, LDH is released. Being an important marker of inflammation, elevated LDH levels may be attributed to endothelial and circulating cells such as platelets and leukocytes [37]. In AIS, inflammation affects the development, progression, and prognosis of the stroke [38]. Several studies have linked LDH to stroke prognosis. For example, elevated LDH levels in cerebrospinal fluid have been proposed as a marker of early brain injury in subarachnoid hemorrhage-related cerebral ischemia [39]. Jin et al. reported that AIS patients with high LDH levels had a higher risk of stroke recurrence and death during an 18-month follow-up, demonstrating its association with stroke severity and poor prognosis [40].

Each of these three parameters holds prognostic value in acute ischemic stroke (AIS). We believe that EASIX, by integrating these variables into a single index, provides improved prognostic utility. The findings of the present study demonstrate a significant association between EASIX and patient survival.

Treatment and follow-up of ischemic stroke vary according to the etiology. There was insufficient data to support a strong distinction between lacunar and non-lacunar stroke for other biomarkers. However, endothelial biomarker expression varies by vessel size (e.g., capillary vs. arteriole/large artery endothelium), which may obscure distinctions between non-lacunar and lacunar strokes [6,41]. Kleeberg et al. suggested that EASIX, reflecting systemic endothelial dysfunction independent of tissue type, could address this limitation [19]. In a study of acute myocardial infarction (AMI) patients, a relationship between EASIX and MI types (ST-MI, non-ST-MI), as well as gender, was reported [26]. In our study, no significant relationship was found between EASIX and gender or TOAST subtypes (embolic, lacunar, and other etiologies). This finding may be related to the relatively small sample size, limited to patients with large-vessel occlusion, or to the fact that EASIX represents a tissue-independent biomarker.

Experimental studies in rodents have demonstrated that hyperlipidemia exacerbates ischemic injury by promoting endothelial cell damage, oxidative stress, inflammation, and neuronal death [42]. Collectively, these parameters reflect endothelial stress, which contributes to reperfusion injury, thromboinflammation, and ultimately poor clinical outcomes in patients with PCIS.

Hyperlipidemia has also been shown to negatively influence outcomes in patients undergoing reperfusion therapies such as intravenous thrombolysis (tPA) and mechanical thrombectomy (MT) [43]. The higher frequency of hyperlipidemia among patients with elevated EASIX values may reflect its contribution to endothelial dysfunction. However, data on hyperlipidemia in relation to EASIX remain limited, underscoring the need for further investigation.

Aging is another key factor linked to endothelial dysfunction in both cardiovascular disease and stroke. With advancing age, endothelial and vascular smooth muscle cells develop functional impairments, and alterations occur in intracellular signaling and endothelial–smooth muscle communication [44]. The higher EASIX values observed in patients aged over 70 years in our study further support the relationship between age-related endothelial dysfunction and poor stroke prognosis [45].

The NIHSS is a validated and widely used tool for evaluating stroke severity in patients with acute ischemic stroke (AIS). Higher NIHSS scores indicate more severe neurological impairment. In our study, patients with elevated EASIX values also exhibited higher 24 h NIHSS scores, supporting the prognostic significance of EASIX. Although intubation (particularly lasting > 3 days), post-reperfusion hemorrhage, APACHE II > 13, and admission NIHSS > 12 were each associated with increased mortality risk, no direct relationship was observed between these variables and EASIX. Future multicenter studies with larger cohorts may further clarify the predictive role of EASIX in determining intubation needs, ventilation duration, and length of intensive care unit stay.

In a previous study evaluating endothelial dysfunction biomarkers (e.g., von Willebrand factor [vWF], E-selectin, vascular cell adhesion molecule-1, and intercellular adhesion molecule-1) in patients undergoing mechanical thrombectomy (MT), elevated levels of these markers were correlated with the occurrence of parenchymal hematoma [46]. In contrast, our study did not demonstrate a significant association between EASIX and post-MT parenchymal hemorrhage, likely due to the limited sample size and the small number of hemorrhagic cases. Further research is warranted to investigate the potential relationship between EASIX and parenchymal hematoma following reperfusion therapy.

Our findings indicate that higher EASIX values may predict poorer prognosis in patients with posterior circulation ischemic stroke. This association may be mechanistically explained by the components of EASIX: creatinine as a marker of renal and endothelial dysfunction, LDH as an indicator of cellular injury and systemic inflammation, and platelet count as a measure of thrombotic activity and consumption. Collectively, these parameters reflect the degree of endothelial dysfunction, which plays a central role in stroke evolution and clinical outcomes.

Numerous biomarkers have been explored for their prognostic significance in acute ischemic stroke (AIS). Shen et al. demonstrated that elevated NT-proBNP levels were associated with larger ischemic core volumes on computed tomography perfusion (CTP) imaging and poorer 90-day functional outcomes [15]. However, NT-proBNP is limited in clinical practice by issues of cost, specificity, and availability. Similarly, multiple meta-analyses have demonstrated that elevated CRP, including high-sensitivity CRP, is strongly associated with mortality, recurrent stroke, and poor functional outcomes. Chen et al. found that high hs-CRP levels at admission were associated with nearly a four-fold increase in mortality and higher risk of recurrent stroke in AIS patients [47] while Yu et al. showed that CRP elevation independently predicted all-cause mortality after stroke [48].

Although these biomarkers provide valuable prognostic insights, their use in clinical practice is limited by factors such as high cost, restricted availability, and suboptimal specificity. In contrast, the EASIX represents a composite score based on routine laboratory parameters, combining markers of endothelial stress into a single, low-cost, and easily obtainable prognostic tool.

## 5. Conclusions

In this retrospective study, we found that higher EASIX values were significantly associated with increased 90-day mortality in patients with posterior circulation ischemic stroke undergoing reperfusion therapy. These findings suggest that EASIX, calculated from routine laboratory parameters, may serve as a simple, inexpensive, and readily available tool for early risk stratification in this high-risk patient population.

To the best of our knowledge, this is the first study to investigate EASIX in reperfusion-treated posterior circulation ischemic stroke, thereby addressing an important gap in the current literature. From a clinical perspective, patients with elevated EASIX values may warrant closer monitoring and, potentially, more intensive management strategies.Future research should aim to include larger, multicenter, prospective cohorts, incorporate serial EASIX measurements, and evaluate its prognostic relevance not only in posterior circulation stroke but also in anterior circulation and lacunar subtypes. Moreover, the relationship between EASIX and long-term outcomes—such as functional recovery, disability, and recurrent stroke—requires further investigation.

This study has several limitations. Its retrospective, single-center design inherently limits the generalizability of the findings. The relatively small sample size—particularly the limited number of patients in the high-EASIX group (*n* = 9) and the small proportion of patients with good functional outcomes (mRS 0–2, *n* = 21)—reduced the statistical power of both the univariate and multivariable analyses. Although the EASIX showed a significant association with 90-day mortality in the univariate analysis, the low number of events likely limited the ability of the multivariable model to demonstrate an independent predictive effect. Thrombolytic therapy was not associated with mortality in the univariate analysis and was therefore not included in the multivariable logistic regression, minimizing the potential for confounding.

Additionally, although all EASIX components were collected as part of routine clinical care, platelet and creatinine values were obtained at emergency department admission, LDH was measured after the endovascular procedure during intensive care unit (ICU) admission. This non-uniform sampling may have affected the comparability of the parameters.

Furthermore, EASIX was measured only once, preventing the evaluation of temporal changes. The exclusion of patients with systemic inflammatory diseases, malignancy, or hematologic disorders may have improved internal validity by reducing confounding, but may limit external validity. Taken together, these findings should be considered preliminary and hypothesis-generating, and validation in larger, prospective, multicenter studies is warranted.

## Figures and Tables

**Table 1:** Distribution of demographic and clinical characteristics of the participants.

	*n* (%)/M (Q1–Q3)
Sex (*n*)	
Male	43 (74.1)
Female	15 (25.9)
Age (years)	70 (57–76)
EASIX	0.81 (0.58–1.27)
Length of hospital stay (days)	10 (6–24)
NIHSS at admission	12 (8–18)
NIHSS at 24 h	15 (5–23)
Door-to-groin time (min)	102 (70–148)
Symptom-to-groin time (min)	260 (162.5–332.5)
Symptom-to-door time	134.5 (60–240)
Door-to-recanalization time (min)	127 (100.5–175.25)
mRS at discharge	5 (3–6)
mRS at 90 days	5 (2–6)
Intubation	33 (56.9)
Duration of intubation	3 (0–11)
ASPECT	10 (0–10)
APACHE II	13 (8–18)
Heart disease	31 (53.4)
DM	21 (36.2)
Previous stroke	11 (19)
Past TIA	3 (5.2)
Smoking	14 (25)
Alcohol	3 (5.3)
HT	41 (70.7)
Hyperlipidemia	13 (22.4)
AF	24 (42.1)
In-hospital mortality	24 (41.4)
Procedure	
Thrombectomy + tPA	47 (81)
Thrombectomy	11 (19)
TICI	
TICI 0	6 (10.3)
TICI 1	4 (6.9)
TICI 2a	0 (0)
TICI 2b	6 (10.3)
TICI 2c	3 (5.2)
TICI 3	39 (67.2)
TOAST	
Large-artery thrombosis	29 (50)
Cardioembolism	18 (31)
Lacunar	0 (0.0)
Other causes	2 (3.4)
Unknown causes	9 (15.5)
Hemorrhage	13 (22.4)
Presence of hemorrhage symptoms	
Symptomatic	9 (69.2)
Asymptomatic	2 (15.4)
mRS at 90 days (MRS:0–2)	21 (36.8)
Decompression	2 (3.4)
Localization of occlusion	
Vertebral artery	7 (12.1)
Posterior cerebral artery	6 (10.3)
Basilar artery	45 (77.6)

NIHSS: the National Institutes of Health Stroke Scale scores. EASIX: endothelial stress and activation index, mRS: modified Rankin Scale, ASPECT: Alberta Stroke Program Early Computed Tomography Score, APACHE II score: acute physiologic assessment and chronic health evaluation score, Thrombolysis in Cerebral Infarction (TICI): Thrombolysis in Cerebral Infarction Trial of Org 10172 in Acute Stroke Treatment (TOAST): Trial of ORG 10172 in Acute Stroke Treatment Classification, TIA: transient ischemic attack, AF: atrial fibrillation, DM: diabetes mellitus, and HT: hypertension.

**Table 2:** Predicting mortality in patients according to EASIX cut-offs.

Test Result Variables	Cut-Off	AUC (95 CI)	Std. Error	*p*	Sensitivity	Specificity	PPV	NPV
EASIX	>1.63	0.583 (0.428–0.739)	0.08	0.295	0.292	0.942	0.778	0.653

**Table 3:** Comparison of basic clinical features of cases between low- and high-EASIX groups.

	EASIX Group		
	Low EASIX < 1.63 (*n* = 49)	High EASIX > 1.63 (*n* = 9)	
	M[Q1–Q3]/*n* (%)	M[Q1–Q3]/*n* (%)	*p*-Value
Male	38 (77.6%)	5 (55.6%)	0.166
Female	11 (22.4%)	4 (44.4%)	
Age < 70	33 (67.3%)	2 (22.2%)	**0.011**
Age > 70	16 (32.7%)	7 (77.8%)	
Smoking	13 (27.7%)	1 (11.1%)	0.294
Alcohol	3 (6.3%)	0 (0.0%)	0.441
DM	17 (34.7%)	4 (44.4%)	0.576
HT	33 (67.3%)	8 (88.9%)	0.192
Heart disease	24 (49.0%)	7 (77.8%)	0.111
Previous stroke	10 (20.4%)	1 (11.1%)	0.513
Past TIA	3 (6.1%)	0 (0.0%)	0.446
Hyperlipidemia	8 (16.3%)	5 (55.6%)	**0.009**
Wake-up stroke	6 (12.2%)	2 (22.2%)	0.425
Intubation	25 (51.0%)	8 (88.9%)	**0.035**
Procedure			
Thrombectomy	9 (18.4%)	2 (22.2%)	0.786
Thrombectomy + tPA	40 (81.6%)	7 (77.8%)	
TICI			
TICI 0	5 (10.2%)	1 (11.1%)	0.933
TICI 1	3 (6.1%)	1 (11.1%)	
TICI 2b	5 (10.2%)	1 (11.1%)	
TICI 2c	3 (6.1%)	0 (0.0%)	
TICI 3	33 (67.3%)	6 (66.7%)	
TOAST			
Large artery	24 (49.0%)	5 (55.6%)	0.893
Cardioembolic	15 (30.6%)	2 (22.2%)	
Lacunar	0 (0.0%)	0 (0.0%)	
Other causes	2 (6.1%)	0 (0.0%)	
Unknown causes	7 (14.3%)	2 (22.2%)	
Hemorrhage	11 (22.4%)	2 (22.2%)	0.988
MRS at 90 days (MRS:0–2)	17 (35.4%)	4 (44.4%)	0.606
AF	21 (42.9%)	3 (37.5%)	0.776
Decompression	1 (2.0%)	1 (11.1%)	0.170
Localization			
Basilar artery	37 (75.5%)	8 (88.9%)	0.526
Posterior cerebral artery	6 (12.2%)	0 (0.0%)	
Vertebral artery	6 (12.2%)	1 (11.1%)	
In-hospital mortality	17 (34.7%)	7 (77.8%)	**0.016**
NIHSS at admission	12.00 [7.50–18.00]	19.00 [12.00–23.00]	0.105
NIHSS at 24 h	13.50 [4.00–20.00]	24.00 [16.00–26.00]	**0.049**
Door-to-groin time (min)	102.00 [75.00–145.00]	102.00 [70.00–152.00]	0.981
Symptom-to-door time(min)	145.50 [60.00–240.00]	71.00 [25.00–162.00]	0.118
Symptom-to-groin time (min)	260 [163–333]	268 [168–333]	0.925
mRS at discharge	5 [2–6]	6 [5–6]	**0.048**
mRS at 90 days	4.00 [2.00–6.00]	6.00 [6.00–6.00]	**0.048**
Duration of intubation	0.50 [0.00–11.00]	9.00 [3.00–9.00]	0.106
ASPECT	10.00 [0.00–10.00]	10.00 [9.00–10.00]	0.500
APACHE II	12.50 [7.50–18.00]	15.00 [15.00–22.00]	0.181
Glucose at admission (mg/dL)	150.00 [126.00–197.00]	107.00 [101.00–152.00]	0.075
WBC (/L)	11.05 [9.04–13.29]	9.49 [8.23–10.79]	0.144
NEU (/L)	8.69 [6.58–10.62]	7.82 [4.70–8.69]	0.198
LYMP (/L)	1.44 [0.85–2.19]	1.46 [0.62–2.06]	0.577
HGB (mg/dL)	12.80 [11.90–14.70]	13.30 [11.80–13.80]	0.644
PCT (/L)	0.23 [0.21–0.29]	0.26 [0.21–0.31]	0.712
MPV (/L)	10.20 [9.50–11.00]	10.80 [10.15–11.80]	0.138
Glucose (mg/dL)	140.00 [109.00–194.00]	130.00 [110.00–152.00]	0.872
Urea (mg/dL)	25.90 [15.60–34.70]	25.80 [23.10–32.60]	0.548
Creatinine (mg/dL)	0.90 [0.75–1.00]	1.40 [1.10–1.75]	**<0.001**
PLT (/L)	235.00 [211.00–273.00]	234.00 [180.00–265.00]	0.384
T-Chol (mg/dL)	189.00 [169.00–211.00]	174.00 [153.00–185.00]	0.207
LDL-c (mg/dL)	120.00 [96.00–141.00]	104.50 [80.50–121.50]	0.201
HDL-c (mg/dL)	41.50 [36.50–51.00]	39.00 [29.00–46.00]	0.469
TG (mg/dL)	108.00 [77.50–197.50]	154.00 [118.00–167.00]	0.237
LDH (mg/dL)	198.50 [163.50–257.00]	396.00 [310.00–447.00]	0.001

NIHSS: the National Institutes of Health Stroke Scale scores. EASIX: endothelial stress and activation index, mRS: modified Rankin Scale, ASPECT: Alberta Stroke Program Early Computed Tomography Score, APACHE II score: acute physiologic assessment and chronic health evaluation score, TICI: Thrombolysis in Cerebral Infarction, TOAST: Trial of ORG 10172 in Acute Stroke Treatment Classification, TIA: transient ischemic attack, AF: atrial fibrillation, DM: diabetes mellitus, HT: hypertension. Bold values indicate statistical significance (*p* < 0.05).

**Table 4:** Univariate and multivariate logistic regression analysis for mortality.

	Univariate		Multivariate	
	*OR (95 CI)*	*p-Value*	*OR (95 CI)*	*p-Value*
Sex (male)	0.926 (0.280–3.067)	0.900		
Age > 70	1.551 (0.533–4.511)	0.420		
EASIX (High > 1.63)	6.588 (1.230–35.277)	**0.028**	1.108 (0.203–42.99)	0.998
Smoking	1.471 (0.436–4.958)	0.534		
Alcohol	2.909 (0.248–34.087)	0.395		
DM	2.031 (0.683–6.042)	0.203		
HT	1.012 (0.321–3.191)	0.984		
Heart disease	1.051 (0.368–2.996)	0.927		
Previous stroke	0.771 (0.198–2.999)	0.708		
Past TIA	0.696 (0.059–8/139)	0.772		
Hyperlipidemia	0.556 (0.149–2.072)	0.381		
AF	0.600 (0.202–1.786)	0.356		
Wake-up stroke	0.424 (0.078–2.311)	0.321		
Intubation	12.833 (3.167–52.007)	**<0.001**	2.809 (0.001–8.469)	0.997
Procedure (Thrombectomy + tPA)	0.517 (0.138–1.945)	0.329		
TICI (reference: TICI 0)	1	0.223		
TICI 1	3 (0.188–47.963)	0.437		
TICI 2b	2 (0.112–35.807)	0.560		
TICI 2c	2 (0.112–35.807)	0.638		
TICI 3	0.444 (0.078–2.529)	0.361		
TOAST (reference: large-artery thrombosis)	1	0.326		
Cardioembolic	0.330 (0.087–1.255)	0.104		
Lacunar	0.001 (0.001–3.028)	0.999		
Other causes	0.001 (0.001–2.109)	0.999		
Unknown causes	2.143 (0.448–10.255)	0.340		
Hemorrhage	7.381 (1.755–31.038)	**0.006**	7.101 (0.012–14.098)	0.996
mRS at 90 days (mRS: 0–2)	0.067 (0.013–0.333)	**<0.001**		
Decompression	1.435 (0.085–24.131)	0.802		
Localization (reference: BA)	1	0.642		
PCA	0.750 (0.124–4.533)	0.754		
VA	2 (0.399–10.019)	0.399		
NIHSS at admission > 12	3.5 (1.158–10.578)	**0.026**		
NIHSS (24.) > 15	6.571 (1.983–21.782)	**0.002**	1 (0.038–25.980)	0.999
Door-to-groin time (min) > 102	1.143 (0.390–3.347)	0.808		
Symptom-to-door time (min) > 134.5	0.615 (0.201–1.887)	0.396		
Door-to-recanalization time (min) > 127	1.57 (0.53–4.59)	0.414		
mRS score at discharge > 5	125.4 (13.620–1154.584)	**<0.001**		
mRS at 90 days > 5	82.5 (13.853–491.325)	**<0.001**	2.346 (0.001–13.989)	0.998
Duration of intubation (days) > 3	6.133 (1.853–20.291)	**0.003**	0.001 (0.001–8.989)	0.997
ASPECT: 10	0.706 (0.498–1.234)	0.191		
APACHE II > 13	11.875 (3.346–42.142)	**<0.001**	0.948 (0.024–37.627)	0.977
Glucose at admission (mg/dL) > 147.5	1 (0.352–2.844)	0.999		
Symptom-to-groin time (min) > 260	0.393 (0.131–1.182)	0.096		
WBC > 10.84	1 (0.332–2.844)	0.990		
NEU > 8.64	0.752 (0.264–2.145)	0.594		
LYMP > 1.45	0.420 (0.144–1.227)	0.113		
HGB > 12.9	0.564 (0.196–1.622)	0.288		
PCT > 0.23	1.227 (0.425–3.541)	0.705		
MPV > 10.3	0.571 (0.195–1.674)	0.307		
Glucose > 139.5	1.773 (0.616–5.102)	0.288		
Urea > 25.85	1 (0.351–2.278)	0.998		
Creatinine > 0.92	2 (0.692–5.777)	0.200		
PLT > 234.5	1.330 (0.466–3.792)	0.594		
T-chol L > 185	0.536 (0.182–1.581)	0.258		
LDL-c > 119	0.649 (0.215–1.957)	0.443		
HDL-c > 41.5	1 (0.333–3.005)	0.999		
TG > 113	0.796 (0.278–2.285)	0.672		
LDH > 209	1.418 (0.494–4.075)	0.517		

NIHSS: the National Institutes of Health Stroke Scale scores. EASIX: endothelial stress and activation index, mRS: modified Rankin Scale, ASPECT: Alberta Stroke Program Early Computed Tomography Score, APACHE II score: acute physiologic assessment and chronic health evaluation score, TICI: Thrombolysis in Cerebral Infarction, TOAST: Trial of ORG 10172 in Acute Stroke Treatment Classification, TIA: transient ischemic attack, AF: atrial fibrillation, DM: diabetes mellitus, HT: hypertension, PCA: posterior cerebral artery. Bold values indicate statistical significance (*p* < 0.05).

## Data Availability

The original contributions presented in this study are included in the article. Further inquiries can be directed to the corresponding author.
